# Radiation Tolerance and Charge Trapping Enhancement of ALD HfO_2_/Al_2_O_3_ Nanolaminated Dielectrics

**DOI:** 10.3390/ma14040849

**Published:** 2021-02-10

**Authors:** Dencho Spassov, Albena Paskaleva, Elżbieta Guziewicz, Vojkan Davidović, Srboljub Stanković, Snežana Djorić-Veljković, Tzvetan Ivanov, Todor Stanchev, Ninoslav Stojadinović

**Affiliations:** 1 Institute of Solid State Physics, Bulgarian Academy of Sciences, Tzarigradsko Chaussee 72, 1784 Sofia, Bulgaria; d.spassov@issp.bas.bg (D.S.); tsvn@issp.bas.bg (T.I.); stanchev@issp.bas.bg (T.S.); 2 Institute of Physics, Polish Academy of Sciences, Al. Lotników 32/46, 02-668 Warsaw, Poland; guzel@ifpan.edu.pl; 3 Faculty of Electronic Engineering, University of Niš, Aleksandra Medvedeva 14, 18000 Nis, Serbia; vojkan.davidovic@elfak.ni.ac.rs; 4 Institute of Nuclear Sciences "Vinča", University of Belgrade, Mike Petrovića 12-14, 11000 Belgrade, Serbia; srbas@vin.bg.ac.rs; 5 Faculty of Civil Engineering and Architecture, University of Niš, Aleksandra Medvedeva 14, 18000 Nis, Serbia; snezana.djoric.veljkovic@elfak.ni.ac.rs; 6 Department of Technical Sciences, Serbian Academy of Sciences and Arts (SASA), Knez Mihailova 35, 11000 Belgrade, Serbia; ninoslav.stojadinovic@elfak.ni.ac.rs

**Keywords:** radiation hardness, high-*k* dielectrics, charge trapping memories, HfO_2_/Al_2_O_3_ nanolaminates, atomic layer deposition (ALD)

## Abstract

High-*k* dielectric stacks are regarded as a promising information storage media in the Charge Trapping Non-Volatile Memories, which are the most viable alternative to the standard floating gate memory technology. The implementation of high-k materials in real devices requires (among the other investigations) estimation of their radiation hardness. Here we report the effect of gamma radiation (^60^Co source, doses of 10 and 10 kGy) on dielectric properties, memory windows, leakage currents and retention characteristics of nanolaminated HfO_2_/Al_2_O_3_ stacks obtained by atomic layer deposition and its relationship with post-deposition annealing in oxygen and nitrogen ambient. The results reveal that depending on the dose, either increase or reduction of all kinds of electrically active defects (i.e., initial oxide charge, fast and slow interface states) can be observed. Radiation generates oxide charges with a different sign in O_2_ and N_2_ annealed stacks. The results clearly demonstrate a substantial increase in memory windows of the as-grown and oxygen treated stacks resulting from enhancement of the electron trapping. The leakage currents and the retention times of O_2_ annealed stacks are not deteriorated by irradiation, hence these stacks have high radiation tolerance.

## 1. Introduction

The charge trapping in thin dielectric films has been intensively investigated recently in order to employ this phenomenon in the non-volatile memories as a replacement of the existing floating gate technology [1,2,3,4,5,6,7]. The charge trapping memory (CTM) design has a lot in common with the floating gate design. The main difference is that the CTM concept uses charge storage in spatially separated charge traps in dedicated dielectric layers while the floating gate concept relies on keeping charges in a potential well realized through a poly-Si layer (floating gate) sandwiched between two dielectrics [6]. The CTM concept is not new but offers some advantages over the floating gate design that are vital for the continuing scaling of non-volatile memories [7]. The introduction of high-*k* dielectrics in microelectronic technology boosted CTM development as these dielectrics have been proven to possess large densities of traps whose parameters could be tailored by the fabrication processes and consequent treatments. On the other hand, they could be optimized to satisfy the requirements for replacement of SiO_2_ in the nanosized field effect transistors [6,7]. Among the available high-*k* materials, HfO_2_ takes a special place since it is already implemented in real production technologies. The trap densities available in HfO_2_ are adequate for CTM applications [8] but its potential for charge storage media can be further enhanced by its doping/mixing with other metals/oxides. It has been reported that by doping HfO_2_ with Al very promising charge trapping layers can be obtained, although the reasons for the substantial improvements in charge trapping parameters are not clear yet [9,10,11].

The assessment of radiation hardness is an important reliability issue for microelectronic devices as they are used in conditions characterized with increased radiation beyond the natural one-space navigation, radiology equipment, instrumentation for nuclear energy plants and detectors for high-energy physics experiments. The devices in a radiation-intensive environment are subjected to the impact of high-energy particles and / or photons which may cause generation of various electrically active defects, leakage currents, early breakdown or loss of stored information. Despite the intensive investigations carried out recently [12,13,14,15,16,17,18,19,20,21,22,23,24,25,26,27,28,29,30], the radiation response of high-*k*-based metal-insulator-semiconductor (MIS) devices is far less understood compared to metal-SiO_2_-Si (MOS) structures. The radiation hardness of the alternative dielectrics and especially HfO_2_ and Al_2_O_3_ has been evaluated as comparable [12,13] or even better than that of SiO_2_ [14,15] although the average dissipated energy during irradiation is much higher in HfO_2_ than in SiO_2_ layer that results in generation of more electron-hole pairs [16,17]. As with the SiO_2_/Si system, irradiation of HfO_2_ and Al_2_O_3_ leads to an appearance of a positive oxide charge [12,13,14,15,18,19,20,21,22]. However, the damage is not regarded as severe [12,13,14,15]. The large density of electron traps present in high-*k* materials is most likely the reason for such an observation. Hence, during the irradiation the enhanced electron trapping balances the hole trapping and other mechanisms of positive charge formation unlike the case of thermal SiO_2_ where the electron trapping is negligible. The increase of the density of interface states *D*_it_ is another possible radiation-induced effect in the MIS devices. For SiO_2_/Si structures radiation-induced *D*_it_ increases with increasing dose, which is related to the release of H^+^ [12]. In the case of Al_2_O_3_ and HfO_2_; however, more complicated behavior is observed. Although some studies show an increase of *D*_it_ with the dose [12,22,23,24], there are also reports that *D*_it_ is not changed noticeably [20,25] and even studies demonstrating an improvement of *D*_it_ values upon irradiation especially at low doses [14,26,27,28]. In contrast to SiO_2_, in the case of HfO_2_ radiation-induced electron traps generation is also reported [25]. The investigations of leakage currents of Al_2_O_3_ and HfO_2_ films after irradiation also produce somehow contradictory results—a deterioration of the current-voltage (*J-V*) characteristics is obtained in [20,21,22,29] while other works do not find substantial radiation-induced change [23,30]. An improvement of the breakdown voltage is reported in [12,23]. It should also be noted that the radiation response of the complex dielectric stacks might be different compared to the dielectrics building the structure [21].

The observed discrepancy in the response of high-*k* dielectrics (and particularly Al_2_O_3_ and HfO_2_) to the radiation is probably related rather to the initial properties of the investigated layers than to the intrinsic properties of the materials themselves. Unlike the active SiO_2_ layer in the MOS devices which is produced only by thermal oxidation of Si, the deposition of high-*k* materials could be performed by several different methods, using a variety of precursors and deposition conditions as well as post-deposition treatments. This inevitably causes dispersion in the dielectric properties, hence different impact of the radiation.

There are only a few works which study the impact of radiation on CTM with high-*k* dielectrics and similarly to the other parameters, radiation is found both to deteriorate [19] and not to affect the memory windows [18]. Additionally, the results in [18] indicate that radiation reduces the already trapped in the dielectric charge which certainly causes a loss of at least a part of the stored information.

Recently, we demonstrated the excellent application capability of Al_2_O_3_/HfO_2_ stacks obtained by atomic layer deposition technique (ALD) as charge trapping media in CTM-based non-volatile memory devices. Moreover, it was established that by tailoring of the stack structure; optimization of Al_2_O_3_ to HfO_2_ ratio; and applying post-deposition annealing (PDA), substantial improvement of Al_2_O_3_/HfO_2_ stacks’ charge storage characteristics could be achieved [31,32]. The results obtained in [11,31,32] and also supported by others [33], demonstrated unequivocally that oxygen annealing substantially stimulates electron trapping in deep traps, thus enhancing charge storage ability of stacks. On the contrary, rapid thermally annealed (RTA) in N_2_ results in a substantial decrease of the memory windows.

In this work, the effects of γ-radiation (^60^Co) on the electrical characteristics and charge trapping of Al_2_O_3_/HfO_2_ nanolaminated stacks deposited on Si by ALD are investigated. The focus is on the properties (memory windows, leakage currents and retention) which are of primary interest for implementation of these structures in non-volatile memories. The influence of post-deposition ambient annealing on radiation response is also examined, as it strongly affects stacks parameters.

## 2. Materials and Methods

Nanolaminated Al_2_O_3_/HfO_2_ stacks were deposited on p-type (100) Si wafers with resistivity of 6 Ω cm by atomic layer deposition (ALD). The investigated stacks consist of five bi-layer blocks, each block containing 30 cycles of HfO_2_ and 10 cycles of Al_2_O_3_ sublayers. The schematic picture of this structure is presented in Supplementary Figure S1. HfO_2_ deposition was realized with tetrakis (dimethylamido) hafnium (TDMA) precursor, and for the Al_2_O_3_ sublayers trimethylaluminum precursor (TMA) was used. In both processes, H_2_O was used as oxidant and the deposition temperature was 135 °C. The stack deposition starts with Al_2_O_3_ process followed by HfO_2_ one. The total thickness of the nanolaminated dielectric structure is 26 nm as evaluated by Woollman M2000D spectral ellipsometer (JA Woolman Co., Lincoln, NE, USA). A part of the samples were rapid thermally annealed (RTA) in oxygen or nitrogen at 800 °C for 1 min. The electrical properties of the stacks were examined on MIS capacitors with Al top (gate) and backside contacts. The square gate electrodes with an area of 10^−4^ cm^2^ were patterned photolithographycally. Two separate sets of capacitors were irradiated using ^60^Co source—one at 10 kGy and the other at 100 kGy (Si). No external voltage was applied to the capacitor’s terminals during the irradiation. The charge trapping in the nanolaminates were examined through capacitance-voltage (*C*-*V*) measurements in a dark chamber at 1 MHz with an Agilent 4980A LCR meter (Keysight Technologies, Santa Rosa, CA, USA). The leakage currents measurements were carried out with a Keithley 236 SMU (Tektronix Inc, Beaverton, OR, USA). The charge trapping characteristics of the stacks were studied by applying to the capacitors negative and positive square voltage pulses *V*_p_ with a duration of 1 s. After each pulse a consecutive *C*-*V* curve was recorded in order to find the shift of the flat-band voltage, *V*_fb_ (Supplementary Figure S2). The retention characteristics of selected memory capacitors were assessed by applying a charging pulse (12 V, 1 s) to introduce a negative or positive charge and a subsequent monitoring of the evolution of *V*_fb_ over time.

## 3. Results and Discussions

### 3.1. Initial Oxide Charges, C-V Hysteresis and Density of Interface States

The initial oxide charge present in the dielectric stacks, *Q*_ox_, is estimated from the flat band voltage of *C*-*V* curves recorded under small applied voltage sweeps (about −3 ÷ 2 V) at which charge injection in the stacks is negligible. *Q*_ox_ has been found to depend strongly on the PDA treatment [31]. *Q*_ox_ of the as-grown layers is positive ~0.7 × 10^12^ cm^−2^, the treatment in O_2_ increases the value of *Q*_ox_ to about 2 × 10^12^ cm^−2^. PDA in nitrogen; however, results in a change of the sign of *Q*_ox_, i.e., for these samples the initial oxide charge is negative ~−1.8 × 10^12^ cm^2^.

**Figure 1:**
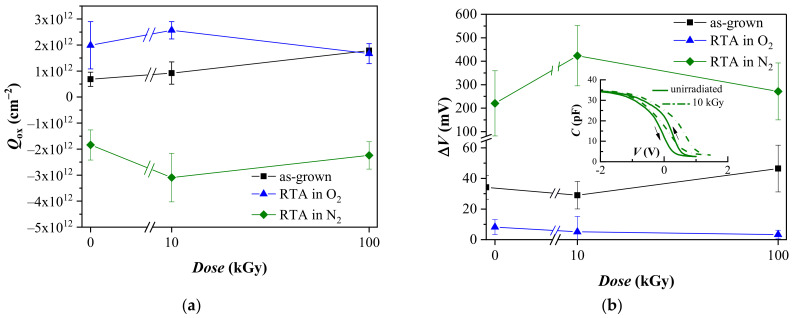
The initial oxide charge (**a**) and *C*-*V* hysteresis (**b**) of stacks before and after γ-irradiation. The inset in (**b**) illustrates the *C*-*V* hysteresis of stacks annealed in N_2_: solid line pristine and dash-dot line after irradiation with 10 kGy.

The radiation response of *Q*_ox_ depends also on PDA (Figure 1a). The generation of a positive oxide charge *Q*_ox_ after irradiation is observed for the as-grown and O_2_ treated stacks. The radiation-induced change in the initial oxide charge is higher for the as-grown samples. 10 kGy exposure slightly increases *Q*_ox_ to about 0.9 × 10^12^ cm^−2^ but for a higher dose, *Q*_ox_ is doubled (~1.8 × 10^12^ cm^−2^). The initial oxide charge of O_2_ annealed stacks also increases to 2.5 × 10^12^ cm^−2^ after 10^4^ Gy irradiation, but further increase of the dose to 100 kGy reduces *Q*_ox_ to the values close to the non-irradiated capacitors. As seen in Figure 1a after 100 kGy irradiation *Q*_ox_ of as-grown samples is equal to the *Q*_ox_ values of O_2_ annealed ones in contrast to the non-irradiated capacitors for which substantially larger *Q*_ox_ is found after O_2_ annealing. The irradiation of stacks treated in N_2_ inflicts appearance of additional negative charges. As with the oxygen treated samples, after the initial increase to about −3 × 10^12^ cm^−2^ for 10 kGy exposure, the higher dose of 100 kGy reduces *Q*_ox_ to its initial non-irradiated values. Therefore, the higher doses seem to inflict a turn-around point in the sign of radiation-generated charge in the annealed stacks. The different sign of the radiation created charge in O_2_ and N_2_ annealed stacks strongly suggests that the nature of the centers giving rise to oxide charge in the two cases is also different.

A small counterclockwise hysteresis Δ*V*^0^_fb_ is observed in the initial *C*-*V* curves measured in a sweep voltage range in which the charge trapping into the stacks is negligible. Δ*V*^0^_fb_ is 34; 8; and 250 mV for the as-grown samples, O_2_ and N_2_ annealed ones, respectively. The hysteresis is usually ascribed to the charge capture at slow states—i.e., traps inside the dielectric located within a tunneling distance from Si. The obtained values for non-irradiated films suggest that PDA in N_2_ creates a substantial number of traps in the first Al_2_O_3_ sublayer, in the possible interfacial SiO_x_ layer and/or at their interface. Generally, γ-radiation changes only slightly the hysteresis of the as-grown and O_2_ annealed stacks, (Figure 1b). The induced changes, however, are very small and close to the detection limits especially in the case of the O_2_ annealed samples, so that tracing a certain tendency cannot be done. The nitrogen treated samples show almost twofold increase of Δ*V*^0^_fb_ to 420 mV after 10 kGy exposure. However, after the 100 kGy exposure Δ*V*^0^_fb_ decreases to the values similar to those of the control non-irradiated capacitors. Hence, the dependence of Δ*V*^0^_fb_ on the γ-radiation dose for the N_2_ treated stacks shows the same radiation behavior as the oxide charge and the density of fast interface states (as shown below).

**Figure 2:**
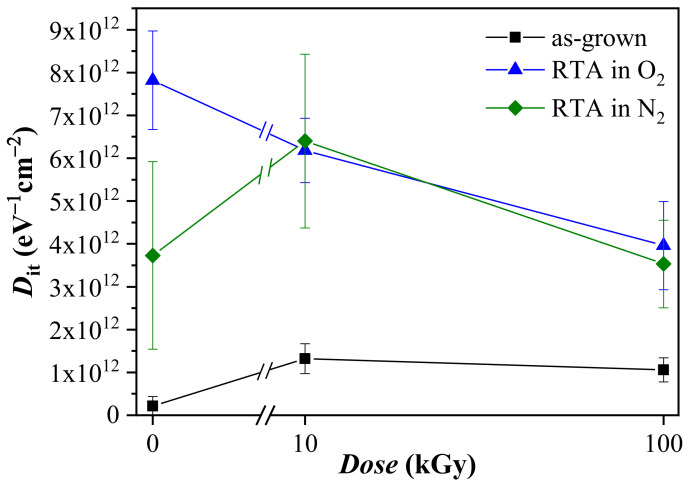
The density of the fast interface states at flat-band before and after irradiation.

The effect of gamma radiation on the density of the fast interface states at Si, *D*_it_, is evaluated through Terman method under the flat-band condition (Figure 2). The choice of the Terman technique over the other more precise methods such as: low-high frequency *C*-*V*; conduction method (*G*—*ω*); and charge pumping seems justified since the fast interface states at Si do not affect directly the charge storage into the stack. The obtained here *D*_it_ serve only as an indication of the radiation hardness of interface between Al_2_O_3_/HfO_2_ stack and Si which thorough study is out of the scope of the present work. Moreover, *D*_it_ is defined by the dielectric at contact with Si and in the real memory cells the charge trapping media is separated from Si by a dedicated tunnel oxide layer (usually SiO_2_). *D*_it_ values of about 2.2 × 10^11^, 7.8 × 10^12^ and 3.9 × 10^12^ eV^−1^ cm^−2^ have been obtained for non-irradiated as-grown, oxygen annealed and nitrogen annealed stacks, respectively. A higher *D*_it_ value for annealed samples indicates that during PDA some kind of interaction between the stack and Si takes place, and oxygen ambient seems to intensify this reaction. The gamma radiation treatment increases density of interface states of the as-grown stacks without a clear dependence on the dose (at 10 kGy the *D*_it_ value is ~1.3 × 10^12^ eV^−1^ cm^−2^ while for 100 kGy *D*_it_ is slightly lower ~1.1 × 10^12^ eV^−1^ cm^−2^). *D*_it_ of the N_2_ annealed layers exhibits a similar behavior–the density of the interface states increases after 10 kGy (to ~6.4 × 10^12^ eV^−1^ cm^−2^), but the 100 kGy irradiation leads to its reduction. However, in this case the decrease is more prominent (twofold reduction) and after the 100 kGy treatment *D*_it_ is almost equal to the values for non-irradiated capacitors. For the oxygen annealed samples *D*_it_ monotonically decreases with the dose and the 100 kGy γ-radiation reduces *D*_it_ almost twice.

The results obtained for the as-grown and oxygen annealed HfO_2_/Al_2_O_3_ stacks corroborate the reports [14,20,23] of higher radiation hardness of HfO_2_ and HfO_2_/Al_2_O_3_-based MOS structures compared to the SiO_2_ based ones. For these structures the effect of radiation mainly consists of a moderate positive oxide charge generation, and in accordance with [14,20,23,26] depending on the dose of γ radiation some improvement of the interface properties (reduction of slow interface states as well as fast interface states in the case of O_2_ annealed samples) of high-*k* stack/Si system can be obtained. At the same time, the data for the N_2_ treated samples suggest that the pre-irradiation processing affects the radiation response, most likely as a result of the different defect structure created under PDA in oxidizing and nitrogen containing ambient. Our previous studies [31] revealed that the amount of negative oxide charge in the N_2_ treated samples depends also on the Al_2_O_3_ content, and the layers with a lower Al_2_O_3_ content exhibit a lower negative *Q*_ox_ or even a positive one for the smallest alumina amount in the stack. In this context, it should be noted that the Al_2_O_3_ layers commonly demonstrate a negative oxide charge [34,35] unlike the HfO_2_ ones whose *Q*_ox_ is predominantly positive. The short annealing in N_2_ [35] is found to increase the negative *Q*_ox_ value which is related to reduction of the density of positively charged Al interstitials, whereas the density of negative O interstitials remains unchanged. Other studies [36] of the effect of nitridation annealing environment (RTN in NH_3_) suggest that the higher negative oxide charge of the HfAlO ALD stacks could be associated with the incorporation of N at the interface between the laminated film and Si. In addition, the N atoms have been found predominantly bonded to Al_2_O_3_, but not to HfO_2_. The N_2_ annealing ambient, however, is regarded as an inert one compared to the case of NH_3_ treatment. Therefore, a more plausible explanation of the observed results is related to the possible high temperature induced transformations in the stacks. The theoretical analysis of defects in the Al-doped HfO_2_ films suggests that under O-rich conditions the most probable defects are electronically compensated–a negatively charged Al ion at the Hf site is compensated by a hole in the valence band. Under oxygen poor conditions the most stable defect is ionically compensated (2Al^−^_hf_) V_O_ (two negative Al ions compensated by a double positively charged neighbor oxygen vacancy). This defect, however, requires two dopant atoms located in a close proximity to each other. Therefore, if the mobility of dopant atoms within the HfO_2_ matrix is low and/or the distance between dopant atoms is large, the formation of this defect might be suppressed. In this case, formation of a mixed compensated Al^−^_hf_ V_O_ defect (negative Al at the Hf site and double positively charged oxygen vacancy V_O_ and electron in conduction band) is fovored. The oxygen poor ambient will likely increase the number of oxygen vacancies in the stack as the formation energy of V_O_ is decreased from 7.5 eV in the O reach conditions to below 2 eV [37]. In fact, some recent investigations [38] notice that the incorporation of aluminum in ALD HfO_2_ films significantly increases the density of oxygen vacancies. Another important aspect of the ALD films is the inevitable presence of hydrogen and C-N radicals as leftovers from the chemical reactions [39,40]. Their interaction with the annealing gas ambient could lead to creation of different types of defects depending on the used PDA environment.

### 3.2. Memory Windows

**Figure 3:**
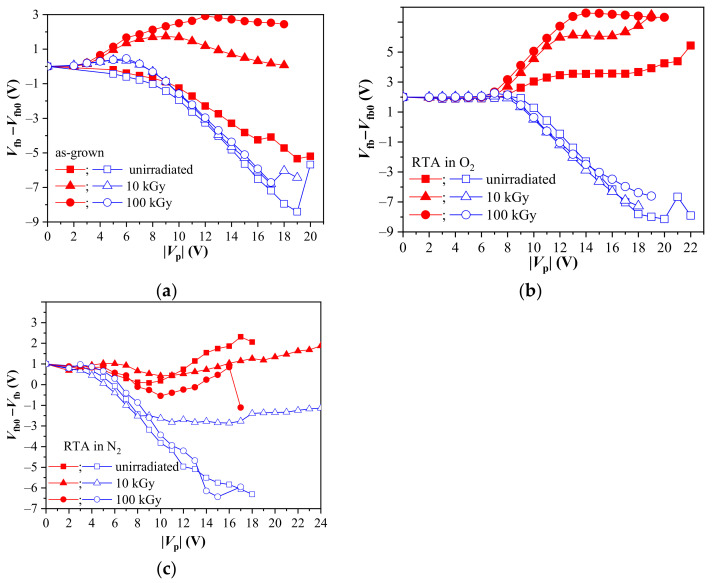
The evolution of the flat-band voltage changes on applying voltage pulses with different *V*_p_, before and after irradiation: (**a**) as-grown; (**b**) O_2_ treated and (**c**) N_2_ treated stacks, respectively. Solid symbols depicts values after +*V*_p_ and hollow after −*V*_p_.

Next, we will consider the effect of γ-radiation on the memory windows of the stacks, formed as a result of the trapping of electrons and/or holes into bulk traps. To get better notion on the different types of charge accumulation processes (electron trapping, hole trapping and/or electric stress-generated defects) contributing to and affecting the memory window, the evolution of the flat-band voltage after applying voltage pulses with duration of 1 s and different amplitude to the capacitors is presented in Figure 3. The data are plotted with respect to the initial (before applying *V*_p_) value of the flat-band voltage, *V_fb0_*. Under positive voltage pulses electrons from the substrate are injected into the stack and their consecutive capturing leads to accumulation of a negative charge into the capacitor. Under negative *V*_p_ holes from Si are injected and trapped into the stack and the emerging charge is positive. (Strictly speaking, for both *V*_p_ polarities the charge carriers with opposite sign with respect to the injected from Si are introduced into the layers from the gate electrode. Therefore, the resulting charge in the dielectric is the sum of trapped carriers supplied by the opposite flows of carries injected from the gate and the substrate. However, as we will see below, the obtained *C*-*V* data follow predominantly the substrate injection scenario). The resulting memory window is defined as the difference between the positions of curves measured at two pulse polarities, along the voltage axis at flat-band point.

As seen in Figure 3a, electron trapping is hardly observed in the as-grown sample before irradiation at |*V*_p_| < 10 V. The *C*-*V* curves for both *V*_p_ polarities are shifted toward negative voltages implying an accumulation of positive charge in the structures. As a result, the memory windows Δ*V* are negligible. Only at |*V*_p_| above 10 V some noticeable electron trapping occurs. With the increase of |*V*_p_| both *C*-*V* characteristics (for negative and positive *V*_p_) are progressively shifted to more negative voltages. Such a behavior is most likely due to the prevalence of the accumulated positive charge over the electron trapping. The increase of positive charge with increasing *V*_p_ under both polarities also implies that some part of it is due to stress-induced positively charged defects, representing irreversible damage. Thus, a net positive charge accretion is observed even at conditions of substrate electron injection. The results, however, could be interpreted also in terms of low initial electron trap density which increases slightly as a result of the stress-related effects, i.e., some of the positively charged defects act as electron traps.

For the samples with PDA in O_2_ before irradiation (Figure 3b) the charge trapping (both positive and negative) is negligible up to |*V*_p_| of 7 V. Unlike the as-deposited stacks, the O_2_ annealed ones show a steady electron trapping which increases at *V*_p_ > 7 V. A noticeable positive charge trapping starts at *V*_p_ about −9 V and for more negative *V*_p_ it increases continuously in the same way as for unannealed samples. Therefore, in the |*V*_p_| range of 7–9 V, the electron trapping prevails. The data clearly indicate that O_2_ annealing creates electron traps which in turn gives rise to a significant memory window (e.g., at *V*_p_ = ±18 V the memory windows, Δ*V*, are 9.5 V and 3.2 V for annealed and as-grown stacks, respectively). The beneficial effect of PDA in oxygen on the memory windows of the ALD Al_2_O_3_/HfO_2_ stacks is also confirmed in [11,33] for structures obtained under different deposition conditions and with different compositions.

The charge trapping for the nitrogen annealed samples before irradiation is negligible at |*V*_p_| below ~5 V. Generally, the positive charge trapping for *V*_p_ above −5 V follows the behavior of the as-grown ones (Figure 3c). At positive *V*_p_ > 5 V, the electron capture is more pronounced than for the as-grown stacks, but the positive charge trapping still prevails. The electron trapping increases more significantly and progressively at *V*_p_ > 10 V, reaching a maximum shift of ~1.5 V at *V*_p_ = 17 V. However, as evidenced in Figure 3b,c, the effects of O_2_ and N_2_ annealing are different, i.e., the gas ambient plays a significant role. The enhanced electron trapping observed for *V*_p_ > 10 V for the N_2_ treated samples could be also associated with a creation of new traps as a result of voltage stress.

The impact of γ-radiation on the charge trapping phenomena in the HfO_2_/Al_2_O_3_ stacks could be summarized as follows:

(1) The positive charge trapping is almost unaffected by irradiation for the as-grown and oxygen treated samples. Indeed, the irradiation slightly decreases the positive charge build-up in the as-grown stacks, and for the O_2_ annealed ones it is somewhat enhanced at *V*_p_ > 10–15 V. Though, the observed effects are small and it can be assumed that irradiation does not generate any new hole traps and does not change the positive charge build-up behavior for these structures. The case of the N_2_ treatment, however, is different. The 10 kGy irradiation slightly increases the positive charge trapping for *V*_p_ < 8 V, but for the higher *V*_p_ the magnitude of the *V*_fb_ shift saturates. For the 100 kGy irradiation the flat-band shift under negative *V*_p_ is almost the same as that for non-irradiated films. Therefore, the behavior of the positive charge build-up at γ exposure seems to correlate somehow with the effect of the irradiation on *Q*_ox_ for these samples. In other words, the high negative *Q*_ox_ at 10 kGy results in the reduced hole trapping, which is restored after the 100 kGy irradiation in accordance with the recovery of *Q*_ox_ to its non-irradiated values.
(2) The data clearly indicate that γ-radiation boosts significantly electron trapping in both the O_2_ annealed stacks and the stacks without PDA. The 10 kGy irradiation of the as-deposited stack results in a noticeable *V*_fb_ shift due to the electron trapping which initially increases with voltage pulse magnitude up to ~9 V, and for higher *V*_p_ the flat-band voltage shift turns-around. Most likely, this kind of dependence is a consequence of interplay between positive charge accumulation and electron trapping at radiation-generated by traps with the domination of the first process. Moreover, a part of the positive charge is probably stress generated, as indicated by the results for non-irradiated stacks [31]. Since the generated electron traps increase with the dose, the turn-around effect for 100 kGy is weakly pronounced and begins at higher *V*_p_. For the O_2_ annealed samples the flat-band voltage shift corresponding to electron trapping tends to saturate at level which increases with the γ-exposure dose. Hence, radiation-induced electron traps play a major role in the charge trapping while the effects of high field stress-induced positive charge are negligible. More complicated response to the radiation is found for stacks annealed in N_2_. The 10 kGy exposure increases electron trapping and negative charge accumulation, respectively up to *V*_p_ = 10 V. For higher *V*_p_, the *V*_fb_ shift is smaller than the observed in non-irradiated layers. The dose of 100 kGy results in the same behavior of the flat-band voltage shift as for the pristine stacks, but with reduced electron trapping. The *V*_fb_ shift corresponding to the electron trapping for the N_2_ treated samples before and after irradiation exhibits a drop at about 9–10 V. This drop is the smallest for the 10 kGy dose and its location coincides with the beginning of the saturation of the positive charge build-up. Thus, the decrease of the electron related *V*_fb_ shift can be attributed to the electric stress-generated positive charge. With the further increase of electric field new electron traps might be created leading as well to an increase of the *V*_fb_ shift under positive *V*_p_.
(3) Because of the intensified electron trapping, the as-deposited and O_2_ annealed stacks demonstrate improved memory windows Δ*V* after irradiation. It is illustrated in Table 1, where Δ*V* values measured at |*V*_p_| = 15 V are given. As is seen, the radiation-induced enhancement of the memory windows is larger for the as-grown films which is a result of a very weak electron trapping in the non-irradiated stacks. Since the irradiation (especially at higher doses) tends to level out the memory windows of the as-deposited and O_2_ annealed stacks, it might be suggested that the traps induced by oxygen annealing and by radiation have the same origin. This is further supported by the radiation response of the N_2_ annealed samples for which γ-radiation shrinks the memory windows due to the reduced electron trapping (and also hole trapping at 10 kGy). The behavior of these samples suggests that the defects developed by PDA in N_2_ are different compared to the as-grown and O_2_ annealed layers.

### 3.3. Leakage Currents and Conduction Mechanisms

**Figure 4:**
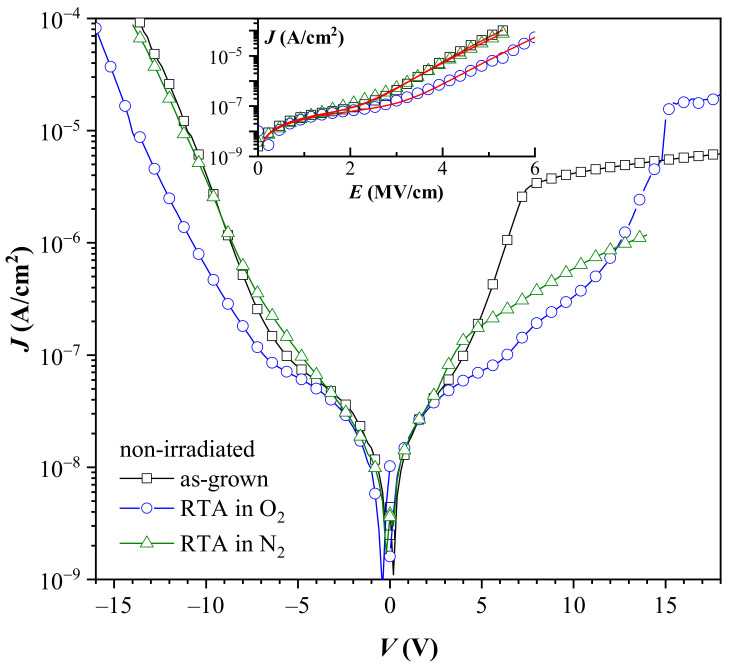
Leakage currents of the non-irradiated stacks. Inset–experimental data for negative applied voltages fitted with a combination of Ohmic and PF conduction (solid lines).

Leakage currents of non-irradiated stacks are depicted in Figure 4.

The annealing procedure does not affect the leakage current values of non-irradiated capacitors in the voltage range of ±5 V. However, for higher applied *V*, annealing influences the leakage. The lowest leakage at high field is observed for PDA in O_2_. The extent of the reduction compared to the as-grown stacks increases with the increase of the electric field and at ±10 V it is larger than 1 order of magnitude. A similar improvement of the leakage currents by oxygen annealing of the HfO_2_-based stacks is also observed in [41,42]. The N_2_ annealing effect on *J* is more unclear. PDA in nitrogen is often reported as a beneficial step for leakage current improvement (in some cases substantial) of ALD HfO_2_ and Al_2_O_3_/HfO_2_ nanolaminated dielectrics [43,44,45,46]. Our results show that PDA affects *J*-*V* characteristics differently, depending on the voltage polarity: for negative *V*, *J* is practically not changed while for positive *V* > +5 V nitrogen processing provides a lower leakage, which values are close to those observed for PDA in O_2_. Considering the effect of PDA on *J* it should be mentioned that divergent effects of PDA on *J* found in the literature seem to be closely related to the initial properties of the layers depending on the parameters of implemented ALD process. Hence, the response of *J* to the PDA could be different for each particular case.

Furthermore, we note that the leakage current does not appear to correlate with the initial oxide charges. Despite the different amount and sign of *Q*_ox_ all structures have similar *J* in the low voltage range (up to ± 5 V). At higher fields the layers with the highest positive initial charge (annealed in O_2_) exhibit the lowest *J*, while as-grown stacks with positive *Q*_ox_ demonstrate leakage currents at negative *V* identical to the N_2_ treated stacks which have negative initial charge. The values of *J* for samples with RTA in O_2_ are comparable to *J* of the nitrogen annealed ones for the positive applied voltages. This behavior might indicate that leakage is governed by bulk-limited conduction mechanisms. (The saturation tendency of *J* at high positive applied *V* is related to the limited amount of minority carriers in p-Si, whereas the slight asymmetry of *J* branches at negative and positive *V* reflects the influence of different barrier heights at gate (Al/HfO_2_) and substrate (Al_2_O_3_/Si) interfaces.) Indeed, the leakage current of the stacks is described reasonably well by a combination of Ohmic (*J*_Ohm_) and Poole-Frenkel (*J*_PF_) conduction (inset in Figure 4):

*J* = *J*_Ohm_ + *J*_PF_ (1)

with:

*J*_Ohm_ = σ_Ohm_·*E* (2)

**Figure 5:**
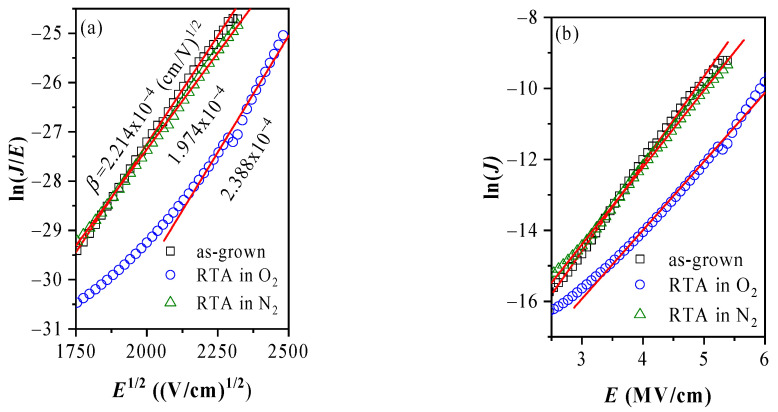
Poole-Frenkel (**a**) and Poole (**b**) plots of *J*-*V* characteristics presented in Figure 4. The values of *β* are calculated for *r* = 1.

*J*_PF_ = σ_PF_·*E*·*exp*(*β*·*E*^1/2^/*r*·k·*T*), *β* = (*q*^3^/*π*·*ε*_0_·*ε*_r_)^1/2^ (3)

where *E* is the electric field, *σ*_Ohm_, *σ*_PF_ are conductivity constants, *q*-electron charge, k-Boltzmann constant, *T*–temperature, *ε*_r_–optical dielectric constant of the stack (*ε*_r_ is square of the refractive index), *r* is a parameter (1 ≤ *r* ≤ 2) describing the presence of additional traps in the dielectric, apart from the PF emitting donor-like center [47,48,49]. *J* has been modeled only for negative *V* for which the Si substrate is in accumulation since in this case the whole voltage drop is on the dielectric itself. The conduction at electric field values below about 3 MV/cm shows the Ohmic-like behavior, while at higher fields it could be modeled by PF expression (inset of Figure 4). The obtained values of *r* and *ε*_r_ are well in the self-consistent range for the investigated stacks (*r* = 1.8 *ε*_r_ = 3.6 for the as-grown; *r* = 1.89, *ε*_r_ = 3.9 for the O_2_ annealed stacks and *r* = 1.95, *ε*_r_ = 3.6 for the N_2_ treated layers). Although *ε*_r_ of nanolaminated HfO_2_/Al_2_O_3_ stack is not known, the obtained from the fit values are close to the value (3.3) estimated by effective media approximation [50] (values of refractive index of HfO_2_ and Al_2_O_3_-1.88 and 1.62 at 5000 nm, respectively [51,52]). (Please note that the agreement between the obtained from Equation (3) high frequency (optical) dielectric constant, *ε*_r_, and its value established from refractive index measurements is assumed as the main indication for the operation of PF mechanism [47,48,49,53,54]). The increase of *ε*_r_ of the annealed stacks certainly reflects the densification of the films after PDA. A closer look to Figure 4 also reveals that *J*-*V* curves for the negative applied voltages demonstrate good linearity in log(*J*) vs. *V* plot scale. This *J*-*V* dependence is characteristic for Poole hopping conduction which variant is the PF mechanism. The current density in Poole conduction is given by:

*J* = *J*_PO_·*exp*[−(*ϕ*_a_ – *q·l·E*/2)/k·*T*] (4)

where *l* is the distance between the adjacent traps, *ϕ*_a_ is the ionization barrier, and *J*_PO_ is a proportionality constant [53]. Therefore, it turns out as demonstrated in Figure 5, that current-voltage curves can be represented by either PF or Poole mechanism in the high field range *E* > 3 MV/cm. (Here we will note that the slopes (*β*) found from the linear fit of the data in Figure 5a (PF scale) are slightly different from the slopes which would be obtained using *ε*_r_ and *r* produced by the fit of *J*-*E* curves to Equation (1).) That is because of the effect of the linear part in Equation (1) (namely Equation (2)). In [53] De Salvo et al., by using a two-trap center model, show that for a certain range of distances between traps, experimental curves are equally well fitted by PF and Poole equations. Following the analysis in [53] the distance between traps estimated assuming *ε*_r_ = 3.3 is as follows: *l* = 1.05, 1.16, and 0.92 nm for the as-grown, O_2_ and N_2_ treated structures, respectively. The estimated trap distances suggest that O_2_ annealing reduces the density of trap-centers taking part in the conduction process. Therefore, one of the reasons for the better charge trapping properties of oxygen treated stacks may be the reduction of the leakage through these layers leading to more efficient trapping of the carriers injected into the dielectric. At the same time, the *J*-*V* data could be also interpreted in the light of some structural transformation of the existing trap levels making them deeper; or generation of new deeper centers; or annealing of the per-existing ones as a consequence of oxygen annealing.

**Figure 6:**
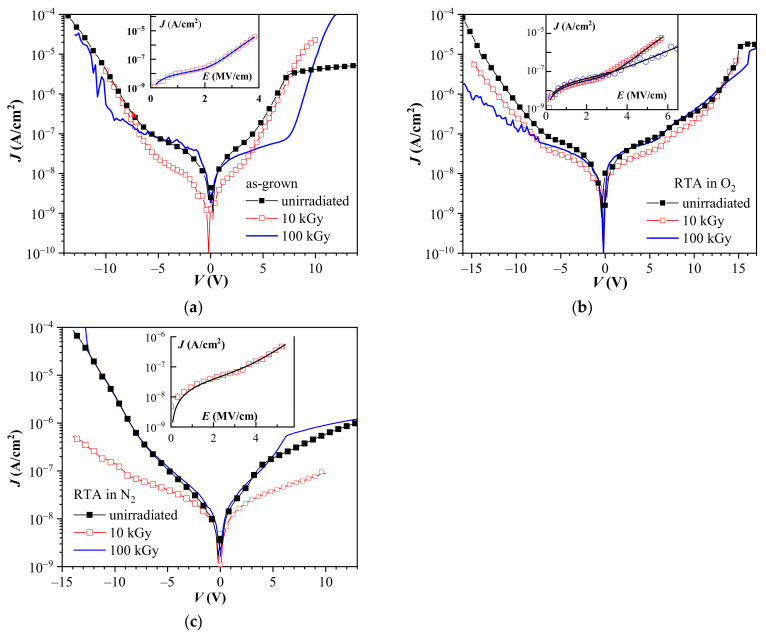
The impact of gamma radiation on the leakage current characteristics of as-grown (**a**) annealed in O_2_; (**b**) annealed in N_2_; (**c**) stacks. The insets represent fits of the experimental data to Equation (1) for 10 kGy (**a**) 10 and 100 kGy (**b**) 10 kGy (**c**).

The effect of gamma irradiation on leakage currents is presented in Figure 6. As shown, the radiation does not lead to an increase of the leakage current unlike the data reported in [20,21]. The results presented here seem to be more consistent with the findings published in [23], where no current deterioration is found. In fact, the irradiation with 10 kGy leads to a lower *J* for all stacks. The induced changes are most prominent for the N_2_ annealed samples (Figure 6c)–for both polarities the current after 10 kGy irradiation is substantially reduced. For the as-grown stacks *J* is reduced mainly in the low electric field of Ohmic-like region while at higher *E* the values of *J* are not affected; the changes are more pronounced for −*V*. In case of oxygen treated stacks *J*-*E* curves after 10 kGy are shifted toward lower *J* values in the whole range of the applied *V* except for the high positive biases. As with the as-grown layers, the current decrease is clearer for the negative applied *V*. For both types of PDA the difference between pristine and 10 kGy irradiated curves increases with the magnitude of the applied negative *V*.

The impact of the 100 kGy dose is more complicated. For the as-grown stacks the values of *J* at negative biases are close to those for non-irradiated structures, except for the voltage interval (−11 ÷ −6 V) in which *J* after 100 kGy is lower. However, at positive applied voltages, the 100 kGy treatment lowers *J* significantly for *V* > 1 V by shifting the curve to higher *V*. Oxygen annealed stacks exhibit further lowering of *J* after the 100 kGy irradiation at high negative *V*, but for the positive applied biases, *J*-*V* curves of non-irradiated and the 100 kGy treated samples are almost the same. For both types of stacks the 100 kGy irradiation inflicts some noise appearance at high applied *V*, (more noticeable under negative *V*). The 100 kGy irradiation returns the *J*-*V* curves of the N_2_ annealed structures in the initial state defined by the pristine case. It should be noted that a similar behavior is observed also for the charge trapping characteristics (Figure 3c).

The irradiation does not seem to change the dominant conduction mechanism; the *J*-*E* characteristics are well described by a combination of Ohmic and PF conduction (solid lines in the insets of Figure 6). For the as-grown samples, however, the 100 kGy curve after the Ohmic part cannot be fitted because of the current fluctuations. The obtained *r* and *ε*_r_ are as follows: for the as-grown stacks 1.5 and 3.6 after the 10 kGy; for layers with PDA in O_2_-*r* = 2, *ε*_r_ = 4 and *r* = 2, *ε*_r_ = 10 after 10 and 100 kGy irradiation, respectively; and *r* = 2, *ε*_r_ = 10 for the annealed in nitrogen structures after the 10 kGy. By applying the analysis of De Salvo et al. [53], the distances between adjacent traps after irradiation have been obtained–*l* = 1.2 nm in the case of as-grown layers after the 10 kGy; *l* = 0.83 and 0.5 nm for the samples annealed in oxygen after 10 and 100 kGy, respectively; and 0.5 nm for the 10 kGy irradiated nitrogen annealed stacks. Therefore, the results indicate that radiation increases the trap density into the stacks resulting in a smaller distance between adjacent traps. It should be noted, however, that determined *l* depends on the value of optical dielectric constant of the stacks, which is not known and is supposed to change after PDA and possibly after irradiation.

As seen in Figure 6, radiation reduces the slope of the high voltage part of *J*-*V* curves (negative applied voltages) of the annealed samples. According to the PF theory this corresponds to an increase of *ε*_r_ or *r*, or both which is indeed obtained by the fitting. The variation of *r* is suggested to result from changes in the ratio of PF-centers, compensating traps and the density of free carriers. *R* = 2 corresponds to the presence of either a single donor center, or a combination of deep (below Fermi level) donor and a shallow trap [47]. In the latter case the donor energy is equal to the sum of the real ionization energies of the donor (PF) center and the shallow trap, which leads to a smaller current. The presence of a noticeable amount of deep compensating acceptor-like centers results in *r* = 1 [48]. The radiation with both doses resulted in an increase of *r* from 1.8 to its upper limit of 2 which might be indeed inflicted by a certain change in the defects structure in the stacks. However, drawing of more specific conclusions is hindered, keeping in mind the uncertainties related with the actual value of *ε*_r_, and that in the PF theory involving two defect centers, one and the same slope can be attributed to different defect combinations [47,48]. At the same time, the increase of *ε*_r_ might be interpreted as an irradiation-induced densification of stacks. It also worth noting that in some recent studies [54] involving simulations of the charge transfer through thin high-*k* films, the identification of PF conduction by its “fingerprint” linearity of ln(*J*/*E*) vs. *E*^1/2^ (and its slope) is contested. Finally, we would like to mention that the observed effect of radiation on the leakage currents seems somehow counter-intuitive since the lowering of the leakage current is accompanied by an increased trap density. (A higher density of traps was obtained from the memory windows measurements as well as the interpretation of leakage current considering both PF and Poole mechanisms [53]). We should note that the same is valid for the non-irradiated nanolaminates as well–the lowest *J* is found for the oxygen treated samples for which the electron trapping is the strongest. Apart from the interpretation that different types of centers are involved in the conduction and charge storage, another possible explanation of this observation is the modification of electric field into the stacks by the built up charge. Therefore, a more complicated analysis of the *J*-*V* characteristics based on the modeling of the electric field redistribution during the current measurement procedure is needed. 

### 3.4. Retention Characteristics

**Figure 7:**
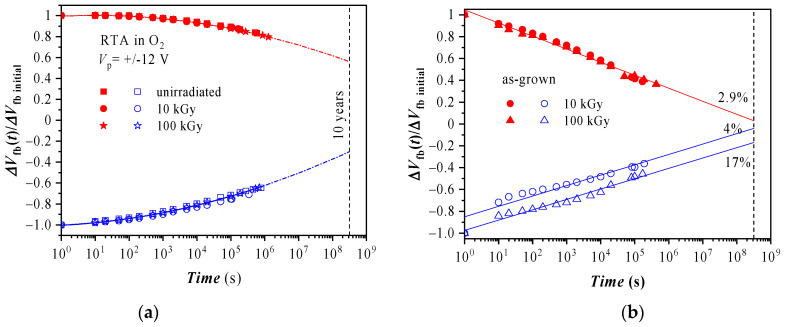
Retention characteristics of stacks annealed in oxygen (**a**) before and after irradiation, and (**b**) irradiated as-grown stacks.

As seen in Section 3.2, the as-grown structures before irradiation as well as the N_2_ annealed Al_2_O_3_/HfO_2_ stacks before and after irradiation provide rather small memory windows due to the weak electron trapping which makes them unattractive from the CTM application point of view. Thus, we will focus mainly on radiation effect on the retention of the O_2_ treated layers (Figure 7a). The retention of as-grown stacks after irradiation is also considered (Figure 7b) in order to answer the question whether the process of radiation-induced enhancement of the trap density could be used in charge storage devices.

The retention is defined as the ratio between Δ*V*_fb_(*t*) (the difference between *V*_fb_ at time (*t*) and *V*_fb_ of uncharged capacitor) to its initial value Δ*V*_fb initial_ immediately after the charging operation. The data clearly indicate that for oxygen treated stacks γ-radiation does not deteriorate the charge retention (Figure 7a). The charge decay with time (*t*) can be well fitted with a *ln^2^(t)* dependence. Such a type of relation between the trapped charge and the retention time is most likely due to domination of Poole-Frenkel discharge currents [31,55]. However, a more detailed analysis, which takes into account the electric field redistribution; possible secondary capture events of emitted electrons/holes; and the charge transfer from/into the electrodes, has to be applied to describe the retention characteristics and to estimate the remnant charge at the 10 years limit. Additionally, the positive charge seems to decline faster than the negative one (Figure 7a). The experimental data show that at 10^6^ s the negative charge remaining in the capacitors is ~80% from its initial value, while the positive charge drops to ~60%. By using *ln*^2^(*t*) dependence for extrapolation to 10 years it is obtained that 44% of the initial negative charge will be lost, and for the positive trapped charge the loss is 70%, i.e., the memory window will be reduced by 57%.

The retention for the as-grown irradiated samples turns out to be independent of the γ-radiation dose especially for trapped electrons. Moreover, the 100 kGy dose seems to improve the retention of the accumulated positive charge. Unlike the case of PDA in O_2_, the discharge in the irradiated as-grown capacitors obeys the exponential dependence on time (linear dependence in semi-logarithmic plot). This kind of dependence is usually observed when the charge loss is realized via tunneling front processes [56]. More elaborated models based on thermal detrapping combined with reduction of the electron escape probability in the volume near the positive-ionized center, left after the first dettrapping act [57] can also explain such a dependence. In contrast to the O_2_ annealed stacks, for as-grown samples the electron discharge rate is faster than the reduction of the positive charge: 12% per decade for electron decay vs. ~10% for the trapped holes. For the 10 kGy exposure case a strong hole detrapping, (a drop of ~25% from the initial charge) is observed within the first 10 s; the 100 kGy irradiation seems to improve to some extend the retention of the accumulated positive charge. It is evident from Figure 7b that the charge loss rate in the case of as-grown films is much higher than for the O_2_ annealed ones. Based on this higher detrapping rate and the difference in the retention characteristics of the as-grown stacks and those with PDA in O_2_, we conclude that radiation-induced traps in as-grown stacks are of different type than the capture centers produced by the oxygen annealing and they are not effective for reliable storage.

## 4. Conclusions

The results clearly demonstrate that ALD HfO_2_/Al_2_O_3_ nanolaminated stacks have good radiation tolerance to γ-rays up to very high doses of 100 kGy. Although γ-irradiation affects the oxide charges and the interfacial properties of the stacks, the inflicted changes are not severe, and depending on the dose, even radiation-induced improvement of the density of fast and slow interface states can be obtained. The specific radiation response of the HfO_2_/Al_2_O_3_ stack depends also on the post-deposition treatment, reflecting the differences in the layer structure after annealing in different ambient. The γ-radiation significantly enhances the electron trapping in the as-grown and oxygen annealed HfO_2_/Al_2_O_3_ nanolaminates due to the creation of new electron traps. As a result, a significant widening of the memory windows is obtained. The nitrogen annealing, however, seems to suppress the generation of radiation-induced electron traps. Generally, the positive charge trapping in all investigated stacks is not affected by the irradiation. No deterioration of the leakage currents and retention characteristics have been observed after irradiation. The obtained results demonstrate that the oxygen annealed HfO_2_/Al_2_O_3_ stacks can be successfully used in CTM devices working in radiation-intensive environment. Moreover, for these stacks γ-treatment with suitable doses can be applied to extend the charge trapping characteristics relevant to the charge trapping-based non-volatile memory devices. In case of the as-grown films, however, the retention times associated with the radiation-generated electron traps are not adequate for application in memory devices, suggesting that radiation-induced traps in oxygen annealed and in as-deposited stacks have different nature.

## Figures and Tables

**Table 1:** Comparison of the memory windows Δ*V* of the investigated stacks as measured at *V*_p_ = ±15 V before and after irradiation.

Dose	Memory Window, Δ*V* (V)		
	As-Deposited	RTA in O_2_	RTA in N_2_
Before Irradiation	1.8	6.8	6.5
10 kGy	5.8	9.7	2.7
100 kGy	7.7	10.6	5.9

## Data Availability

The data presented in this study are available on request from corresponding author.

## Supplementary Materials

The following are available online at https://www.mdpi.com/1996-1944/14/4/849/s1, Figure S1: A schematic presentation of a test wafer with MIS capacitors, blue layers—HfO2, yellow Al2O3, Figure S2: An illustration of the memory window definition. The memory window, ΔV is defined as the difference in the position of the measured C-V curves at flat band capacitance Cfb after applying consecutively positive voltage pulse (+Vp) and negative voltage pulse(–Vp). The inset shows the measurement procedure.
