# Supplementary material for: Radiation Tolerance and Charge Trapping Enhancement of ALD HfO2/Al2O3 Nanolaminated Dielectrics
Source: Materials (Basel). 2021 Feb 10;14(4):849. doi: 10.3390/ma14040849 (PMC7919267; doi:10.3390/ma14040849)
Supplement: Supplementary file 1 [file materials-14-00849-s001.zip › materials-1085546-supplementary.pdf]

materials

MDPI

Supplementary Information

Radiation Tolerance and Charge Trapping Enhancement of ALD HfO_2/Al_2O_3 Nanolaminated Dielectrics

Dencho Spassov, Albena Paskaleva, Elżbieta Guziewicz, Vojkan Davidović, Srboljub Stanković, Snežana Djorić-Veljković, Tzvetan Ivanov, Todor Stanchev and Ninoslav Stojadinović

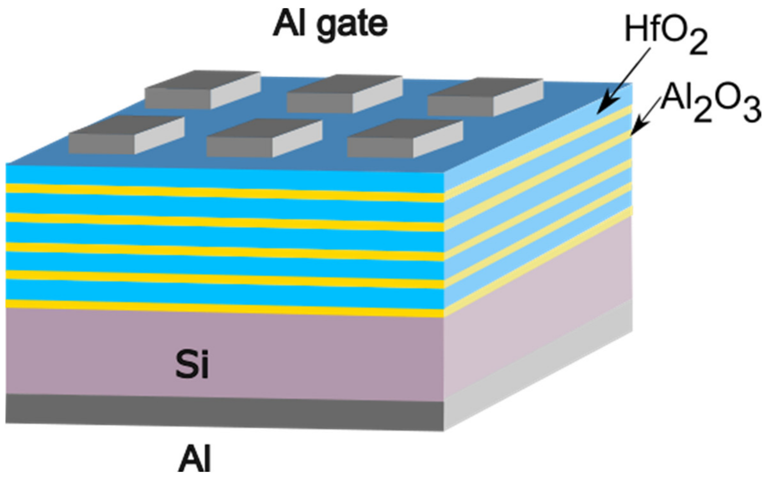

Figure S1. A schematic presentation of a test wafer with MIS capacitors, blue layers—HfO_2, yellow Al_2O_3.

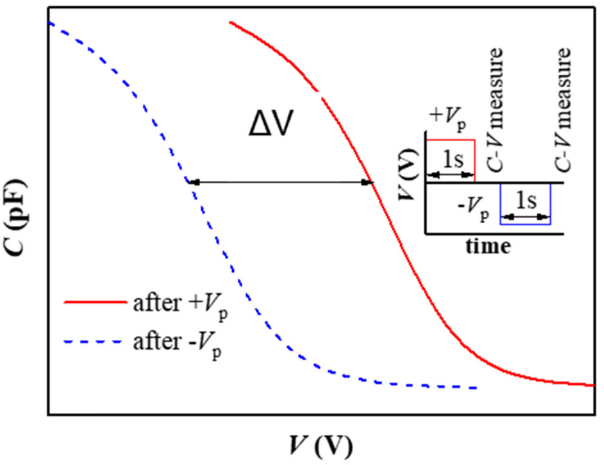

Figure S2. An illustration of the memory window definition. The memory window, Δ*V* is defined as the difference in the position of the measured *C-V* curves at flat band capacitance C_{fb} after applying consecutively positive voltage pulse (+V_p) and negative voltage pulse(−V_p). The inset shows the measurement procedure.

Citation: Spassov, D.; Paskaleva, A.; Guziewicz E.; Davidović V.; Srboljub S.; Djorić-Veljković, S.; Ivanov T.; Stanchev, T.; Stojadinović N. Radiation Tolerance and Charge Trapping Enhancement of ALD HfO2/Al2O3 Nanolaminated Dielectrics. *Materials* **2021**, *14*, 849. https://doi.org/10.3390/ma14040849

Academic Editors: Alain Moissette and Antonio Di Bartolomeo

Received: 6 January 2021
Accepted: 8 February 2021
Published: 10 February 2021

Publisher's Note: MDPI stays neutral with regard to jurisdictional claims in published maps and institutional affiliations.

Copyright: © 2021 by the authors. Licensee MDPI, Basel, Switzerland. This article is an open access article distributed under the terms and conditions of the Creative Commons Attribution (CC BY) license (http://creativecommons.org/licenses/by/4.0/).
